# Staff Appointments at the Cardiff Medical School

**Published:** 1913-11-15

**Authors:** 


### STAFF APPOINTMENTS AT CARDIFF MEDICAL SCHOOL.

A storm in a teacup seems to be raging in Cardiff over the proposed new regulations concerning the qualification of candidates for future appointments to the staff of the King Edward VII.'s Hospital. At the present moment the South Wales University College teaches the preliminary subjects of medical education, but for the clinical studies the students have to go elsewhere. An attempt is to be made to remove this defect and to provide full training for the student right up to complete qualification by converting the Cardiff Hospital from a non-teaching to a teaching institution. From the point of view of the hospital and the patients in it the proposal is clear gain, for it is the universal experience in England that to have a school attached to a hospital inevitably raises the standard of work throughout the various departments. To the Welsh school the advantages of clinical teaching in Cardiff are obvious.

So much is common ground. But dissension arises as to the necessity of insisting on the highest academical standard for future staff appointments. A statute is proposed, to take effect in two years' time, that no one shall be appointed physician unless he be a Fellow or Member of the Royal College of Physicians of London, nor surgeon unless he be a Fellow of the Royal College of Surgeons of England. Those who favour this regulation point out that all the London schools and most of the other English ones enforce it; and that it is necessary not only to the prestige and the efficiency of the new Welsh school, but also to make a favourable impression on the Treasury authorities so as to secure a grant from them. Those who oppose it dwell on the importance of experience as compared with mere academic distinctions, and suspect some intrigue behind the proposal.

It may be taken as undisputed that the new school must aim at nothing less than the best of teaching for its students. It no more follows that a F.R.C.S. or a M.R.C.P. is a good teacher than that he is a brilliant operator or a profound diagnostician. But it is true, in the main, that the soundest teachers, as well as the most scientific and practical clinicians, possess one or other of those qualifications, for the simple reason that those young graduates whose ambition leads them to undertake the long post-graduate study and training essential to a would-be teacher or specialist almost invariably hall-mark their labours, as it were, by the precaution of passing one or other of those examinations. Clinical experience, it is quite true, can only be gained through clinical work; and there are admittedly here and there to be found men who lack these highest academic qualifications, yet are fit to rank with the best as up-to-date and scientific surgeons or physicians. But with teaching it is not so, because in England men do not get any opportunity of teaching students unless they do possess these particular diplomas.

If the Cardiff Hospital wants the best possible medical staff, the proposed regulations will help to secure it; though they may conceivably exclude a good man now and then, they will also exclude far more second-raters. But if the Cardiff school wants the best possible teachers, the proposed regulations are absolutely essential. It will be noticed that no member of the existing staff is to be affected by the new rule; and any who may be appointed before it takes effect need not possess the qualifications in question on election, but must secure them before January 1916. We cannot sympathise with those who oppose this change, for we are convinced that it is salutary.

